# A Genetic Approach to the Recruitment of PRC2 at the *HoxD* Locus

**DOI:** 10.1371/journal.pgen.1003951

**Published:** 2013-11-07

**Authors:** Patrick Schorderet, Nicolas Lonfat, Fabrice Darbellay, Patrick Tschopp, Sandra Gitto, Natalia Soshnikova, Denis Duboule

**Affiliations:** 1 National Research Center ‘Frontiers in Genetics’, Geneva, Switzerland; 2 School of Life Sciences, Federal Institute of Technology (EPFL), Lausanne, Switzerland; 3 Department of Genetics and Evolution, University of Geneva, Sciences III, Geneva, Switzerland; Centre National de la Recherche Scientifique, France

## Abstract

Polycomb group (PcG) proteins are essential for the repression of key factors during early development. In *Drosophila*, the polycomb repressive complexes (PRC) associate with defined polycomb response DNA elements (PREs). In mammals, however, the mechanisms underlying polycomb recruitment at targeted loci are poorly understood. We have used an *in vivo* approach to identify DNA sequences of importance for the proper recruitment of polycomb proteins at the *HoxD* locus. We report that various genomic re-arrangements of the gene cluster do not strongly affect PRC2 recruitment and that relatively small polycomb interacting sequences appear necessary and sufficient to confer polycomb recognition and targeting to ectopic loci. In addition, a high GC content, while not sufficient to recruit PRC2, may help its local spreading. We discuss the importance of PRC2 recruitment over *Hox* gene clusters in embryonic stem cells, for their subsequent coordinated transcriptional activation during development.

## Author Summary

*Hox* genes are essential for the proper organization of structures along the developing vertebrate body axis. These genes must be activated at a precise time and their premature transcription is deleterious to the organism. Early on, *Hox* gene clusters are covered by Polycomb Repressive protein Complexes (PRCs), which help keep these genes silent. However, the mechanism(s) that selectively recruit PRCs to these particular genomic loci remains elusive. We have used a collection of mutant mice carrying a set of deletions inside and outside the *HoxD* cluster to try and detect the presence of any DNA sequence of particular importance in this mechanism. We conclude that a range of low affinity sequences synergize to recruit PRCs over the gene cluster, which makes this process very robust and resistant to genetic perturbations.

## Introduction

Polycomb group (PcG) proteins are essential for proper development of most eukaryotic organisms. The founding member (*Polycomb*) was characterized genetically as a repressor of *Drosophila* homeotic genes [1] and subsequent studies have established these proteins as key organizers of the epigenome (e.g. [2]). For instance, they play important roles in the stable repression of genes *via* epigenetic mechanisms such as X-inactivation [3] and imprinting [4], [5], as well as during cell cycle regulation and differentiation (e.g. [6], [7], [8]). The fine-tuned balance between the activities of PcG proteins and proteins from the *trithorax* families (trxG), displaying opposing functions, was shown to maintain *Hox* gene expression during the entire life of *Drosophila* [9]. However, neither the exact process(es) whereby these proteins impose their repressive effect, nor the specific mechanism(s) involved in the recognition and tethering to target genomic loci are as yet fully understood.

In mammals, PcG proteins are mostly found in two large complexes: the Polycomb Repressive Complexes 1 and 2 (PRC1, PRC2). PRC2 carries a methyl-transferase activity that methylates the histone H3 tail at lysine 27, a mark largely associated with gene silencing. While the initial deposition of this post-transcriptional modification is carried through by PRC2, PRC1 maintains this methylated status and compacts chromatin, largely, though not solely [10], by the ubiquitylation of lysine 119 of histone H2A [11]. It was shown that specific PRC1 type complexes are recruited by PRC2 [12], which can also compact chromatin, though to a lesser extent. The importance of these complexes for various developmental and differentiation processes is reflected by the early lethality induced by the loss-of-function of several of their components such as *Suz12* [13], *Ring1B* [14], *Ezh2* [15] and *Eed* [16]. These components are usually well conserved throughout metazoans, suggesting that both the global operational mode of these complexes, as well as the way they are recruited to specific loci may be comparable between species.

In *Drosophila*, PcG group proteins are recruited to chromatin through the recognition of polycomb response elements (PREs). These DNA segments, which were generally identified by forward genetics, must satisfy three criteria: (1) they must bind polycomb when randomly inserted into the genome, (2) they must be able to induce a H3K27me3 domain and (3) they must repress a reporter construct, when associated with them [17], [18], [19]. *Drosophila* PREs are approximately 1.5 kb long in average and are usually located in proximal promoter regions. This is the case of the PREs associated with either *engrailed* [20], [21], *Hedgehog* [22], [23] or *polyhomeotic* [24], [25].

However, PREs have also been mapped *in vivo* several kilobases away from their target genes and their identification may be biased by genome wide chromatin immunoprecipitation (ChIP) studies. Recently indeed, it has become clear that only a fraction of *polycomb* enriched regions are direct targets of PRC, whereas others are indirect, *via* chromatin looping [26], suggesting that *polycomb* enriched regions may not always reflect the genuine presence of *polycomb* at a particular locus. Instead, they may derive from the three-dimensional organization of the genome, which along with the technology employed, may lead to false positives. Furthermore, although high throughput studies have shown that the binding profiles of *polycomb* proteins correlate with both the transcription start sites (TSSs) and stalled RNA PolII [27], [28], they do not share any salient sequence homology such that no consensus motif has been identified thus far [29].

In mammals, the understanding of the general mechanism (if any) accounting for the recruitment of PRC2 is lacking too. PRC2 recruitment has been associated with the presence of binding sites for the *Pho* ortholog YY1 [30], [31], although the physical interaction between *Pho* and PRC remains controversial [32], [33], [34], [35], [36]. Other candidates include various transcription factor binding sites [37], high density of unmethylated CpG dinucleotide regions or the presence of a TSS [38], [39], [40], [41], [42], [43]. While these explanations apply individually to a range of particular situations, they cannot fully account for the apparent high specificity of PRC2 recruitment genome-wide.

PcG proteins are found over developmental genes in pluripotent embryonic stem (ES) cells, including the four *Hox* gene clusters (Fig. S1 and [44], [45], [46]). In this uncommitted state, a significant fraction of *Pc* target loci also carry *trxG* proteins and their H3K4me3 epigenetic marks. Such ‘bivalent domains’ displaying both H3K27me3 and H3K4me3 modifications are found over genes poised for transcription. During embryonic development, these domains lose either one of the two marks and thus acquire a univalent epigenetic status. In mammals, expression of *Hox* genes in the foremost anterior structures is tightly repressed and, accordingly, H3K4me3 is lost over the four *Hox* clusters, whereas the coverage by *PcG* proteins and H3K27me3 is re-enforced [44]. This emphasizes the necessity, for an organism, to properly and selectively secure the recruitment of PRC2 to the appropriate loci, at the right time.

We have addressed the question of polycomb recruitment at *Hox* loci by using an *in vivo* approach, based on the large number of genomic re-arrangements associated with the *HoxD* locus [47], a DNA region that is amongst the most heavily covered by H3K27me3 marks in ES cells and where one of the few vertebrate PREs has been previously identified [31]. In contrast to what is observed in the *Drosophila* Bithorax complex where a 300 kb large domain of trimethylated histones involves only few PREs, we show that the mouse *HoxD* locus implements a mechanism that can compensate for large and systematic deletions within the target DNA interval. These results indicate that PRC2 recruitment at this locus must rely upon a range of cooperating binding sites, rather than upon a few nucleation sites. By using isolated transgenes, we also show that in this particular context, CpG islands (or a high GC content) are not the prime factors in PRC2 recruitment, despite their potential importance for local spreading.

## Results

### Scanning deletions of the *HoxD* cluster

To study the recruitment of polycomb complexes and the resulting H3K27me3 histone modification *in vivo*, we used a genetic approach of the mouse *HoxD* gene cluster, which is a main target of *Pc* silencing in ES cells and adult tissues [44], [48]. Also, this locus has been shown to contain one of the few defined mammalian PREs [31]. We assessed the binding profiles of different members of the polycomb complexes in both wild type and deletion alleles using chromatin immunoprecipitation (ChIP). Should a given part of this gene cluster be of particular importance for recruiting PRC, its deletion may lead to modifications of the binding of PRC proteins and/or of the general H3K27me3 profile.

**Figure 1:**
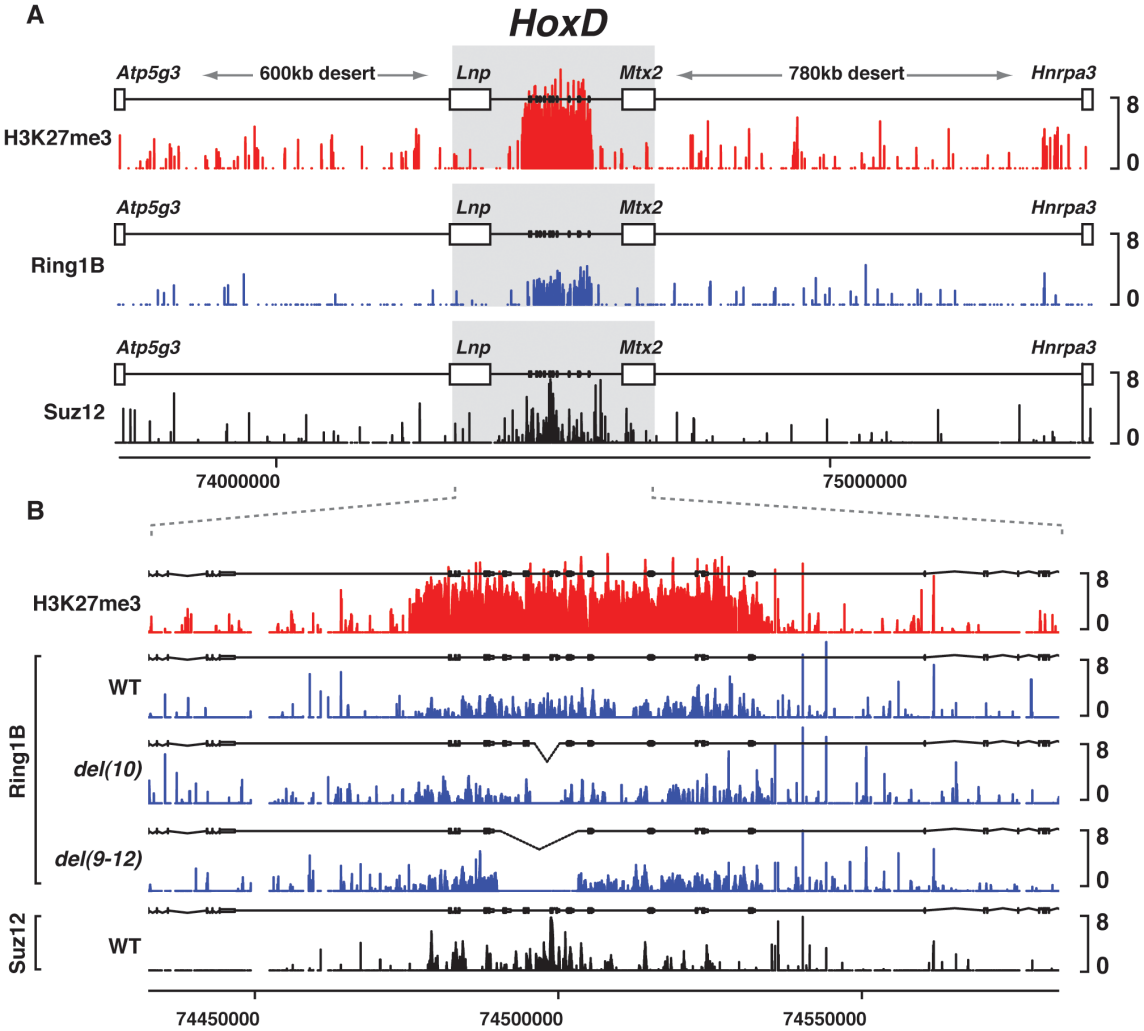
PRC1/2 profiles in various genetic configurations. (A) Wild type genomic landscape of the murine *HoxD* cluster flanked by two large gene deserts on its centromeric and telomeric sides. Log2 profiles of H3K27me3 (red), PRC1 (blue) and PRC2 (black) enrichment obtained on tiling arrays from embryonic tissues. (B) Effect on PRC1 binding profiles of mutant genetic configurations (blue), compared to wild type PRC2 (black) and H3K27me3 (red). Alleles are specified on the left.

**Figure 2:**
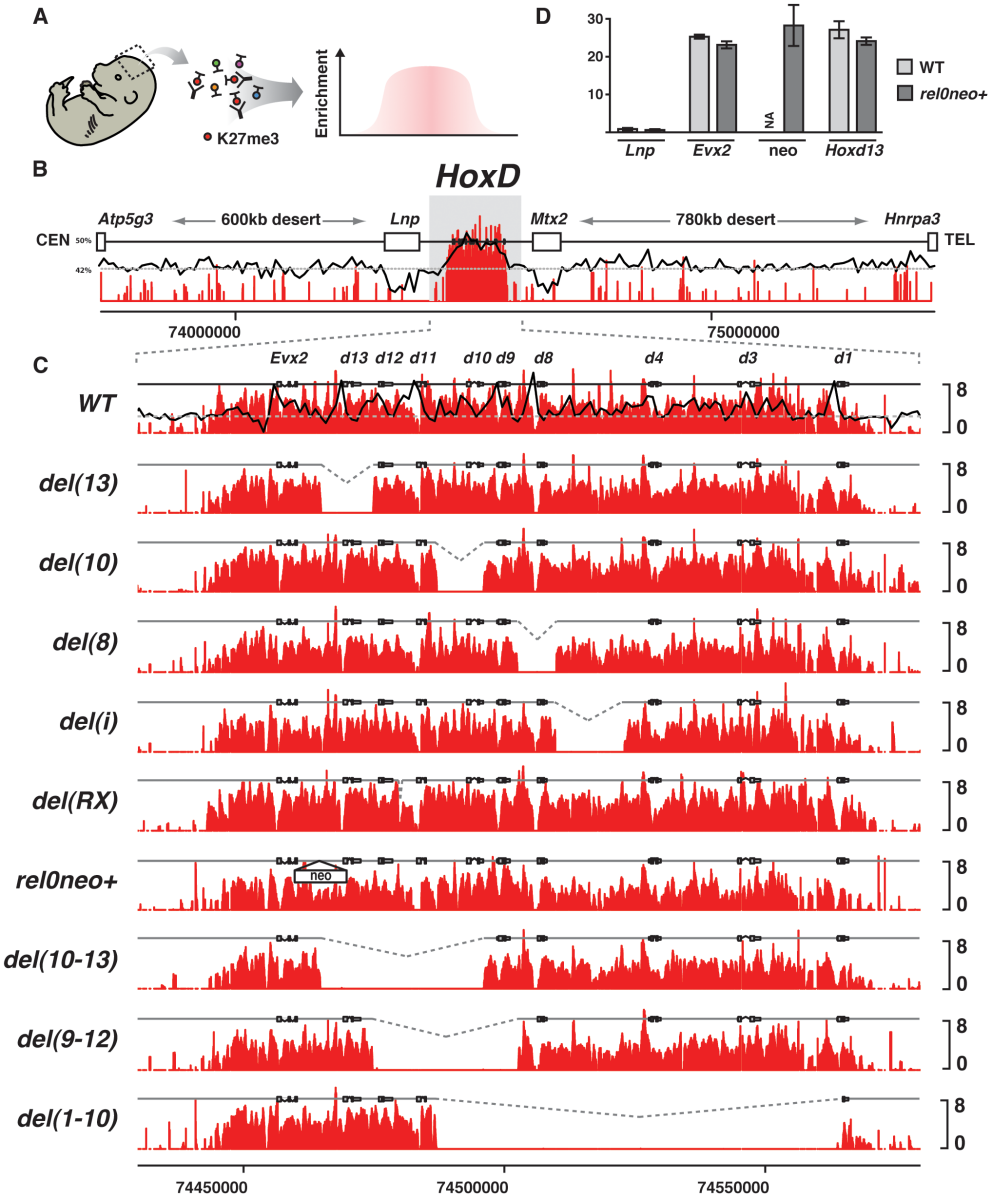
Effect of single *Hox* gene deletions on the H3K27me3 profiles. (A) Schematics of the procedure whereby E13.5 embryonic forebrains are used to ChIP H3K27me3. (B) Wild type genomic landscape of the murine *HoxD* cluster flanked by two large gene deserts on its centromeric (CEN) and telomeric (TEL) sides. The log2 profiles of H3K27me3 enrichment obtained on tiling arrays (red) and GC density using a 10 kb sliding window (black). Dotted grey line corresponds to the average GC content of the mouse genome (42%). (C) H3K27me3 (red) profiles of wild type and various deleted alleles. Genotypes are specified on the left with the extent of the deletion schematized by the dotted lines. GC density using a 1 kb sliding window is depicted in the upper most panel (black line). (D) Comparison between the H3K27me3 profiles of wild type and *rel0neo+* animals as measured by qRT-PCR.

Deletion of parts of the *HoxD* cluster did not seem to affect the binding profiles of PRC proteins when assessed by ChIP-qPCR (Fig. S1). We hybridized the ChIPed material to high density tiling arrays and the overall binding profiles remained largely unchanged throughout the cluster, including those peaks assessed by ChIP-qPCR (Fig. 1A, B). As it was shown that the loss of PRC1/2 leads to the loss of H3K27me3 marks [49] and that H3K27me3 is considered a hallmark of PRC2-mediated gene repression [2], we focused on the analysis of H3K27me3 mark. We used mutant mice carrying complementary deletions covering the entire *HoxD* cluster to try and detect if any of the deleted part would impact upon this epigenetic modification. We performed ChIP of H3K27me3 from E13 embryonic brains (Fig. 2A, B), a tissue where *Hox* genes are both enriched in PRC1 and PRC2 and where *Hox* trancripts are virtually absent. Also the distribution in H3K27me3 marks over *Hox* loci in fetal brain resembles that found in embryonic stem cells [38], [44]. This material was hybridized to tiling arrays covering the mouse *HoxD* cluster [50].

We first evaluated the impact of deleting a small DNA sequence located between *Hoxd11* and *Hoxd12*, which in human cells showed the hallmarks of PREs including the tethering of polycomb proteins and the silencing of an associated reporter gene [31]. This region includes the highly conserved region RX, whose deletion (*del(RX)*) *in vivo* had no effect upon gene expression within the *HoxD* cluster [51], [52], nor did it significantly change the H3K27me3 profile when compared to wild-type animals (Fig. 2C). The PRE reported in this region [31] is slightly larger than region X itself and essential sequences may not have been included in this short deletion. We thus looked at a deletion removing the entire PRE as well as some flanking DNA (Fig. S2; *del(12)*). Here again, we did not score any modification of the H3K27me3 profile throughout the gene cluster. We next scanned the entire locus with a set of adjacent deletions, including either *Hoxd8*, *Hoxd9*, *Hoxd10*, *Hoxd11*, *Hoxd12* or *Hoxd13* and found no significant change in *polycomb*-mediated silencing (data not shown), using H3K27me3 as a proxy for PcG occupancy (Fig. 2C and Fig. S2). Altogether, and in agreement with results obtained in *Drosophila* [53], our data suggest that PRC2 recruitment to mammalian *Hox* clusters does not rely upon few strong PREs, whose activities would then further spread over the rest of the locus.

### Combined deletions

As PREs could be formed by the addition of several low affinity sites, we analyzed deletions of several contiguous genes. The profiles remained surprisingly unmodified, as illustrated by *Del(10-13)* and *Del(9-12)* (Fig. 2C). These two deletions were of particular interest since they cover both the previously described PRE [31] and the region de-repressed in the absence of the LncRNA HOTAIR, which was proposed to bring PRC2 over the *HOXD* cluster [48]. Yet they did not change the H3K27me3 coverage, neither in the *Evx2* locus, nor over the rest of *HoxD*. Other deletions involving several genes in *cis* gave the same result, with no obvious variation in the profiles of H3K27me3 (Fig. 2C and Fig. S2), suggesting that the mechanism recruiting Pc proteins over this locus is robust and can compensate for drastic genomic re-arrangements.

We next asked whether the extremities of the *Hox* gene cluster were of particular importance to set up a platform for recruiting PRC2. We used a large deletion (*del(1-10)*), where two-thirds of the anterior part of the cluster were removed including its most anterior gene *Hoxd1*. Again, the remaining mini-cluster was able to compensate for this significant trimming and the H3K27me3 pattern over the remaining loci was nearly identical to that found in wild type conditions (Fig. 2C). In fact, even the deletion of the entire *HoxD* cluster, from *Hoxd1* to *Hoxd13* (*del(1-13)d11Lac*), did not significantly affect the presence of these epigenetic marks over the remaining 5′ located *Evx2* gene (Fig. S2). In this case, interestingly, the H3K27me3 profile covering the *Evx2* region was similar, in terms of relative peak intensities, to that observed with the shorter *del(4-13)*, even though the latter displayed massive amounts of H3K27me3 over the anterior part of the cluster (from *Hoxd1* to *Hoxd3*; Fig. S2).

This result indicated that each piece of the gene cluster is rather independent in its ability to recruit PRC2, regardless of what would happen over the neighboring loci. The poor impact of the neighboring sequences upon the coverage of any given *Hox* gene loci by H3K27me3 was confirmed by the comparative analysis of *del(10)*, *del(13)* and *del(10-13)*, which share the same breakpoints but in various configurations. In this set of deletions, the reconstitution of three different neighborhoods did not modify the methylation patterns.

### Influence of intergenic distances on H3K27me3 coverage

Single gene deletions did not markedly change the relative distance between transcription units and hence they may not affect PRC2 recruitment, should several PREs locate near each gene and synergize. We thus modified the distance between two genes by excising the longest gene free DNA segment within *HoxD*. This intergenic region ‘*i*’ is over 13 kb long and maps between *Hoxd4* and *Hoxd8*. Its deletion (*del(i)*) results in a further concentration of genes by bringing *Hoxd1*, *Hoxd3* and *Hoxd4* closer to the centromeric (posterior) side including *Hoxd8* to *Hoxd13*. *del(i)* did not show any clear difference in the H3K27me3 profile over the remaining parts of the gene cluster (Fig. 2C, *del(i)*).

We next looked at the effect of introducing a gene free region within the cluster such as to increase the distance between neighboring genes. We duplicated region *i* to produce a cluster with a 26 kb large gene-free domain inside. Intriguingly, the duplicated configuration did not exhibit any change on region *i*, except for a weak gain of signal over the duplicated region suggesting that both copies are covered by H3K27me3. The flanking DNA segments, however, displayed the same H3K27me3 profiles as in wild type brains (Fig. 2C and Fig. S2, *del(i)*, *dup(i)*) indicating that the mechanism recruiting PRC2 over the mouse *HoxD* cluster can compensate for modifications in the distance between transcription units, emphasizing once more the robustness of this process.

### Epigenetic borders and spreading of H3K27me3

**Figure 3:**
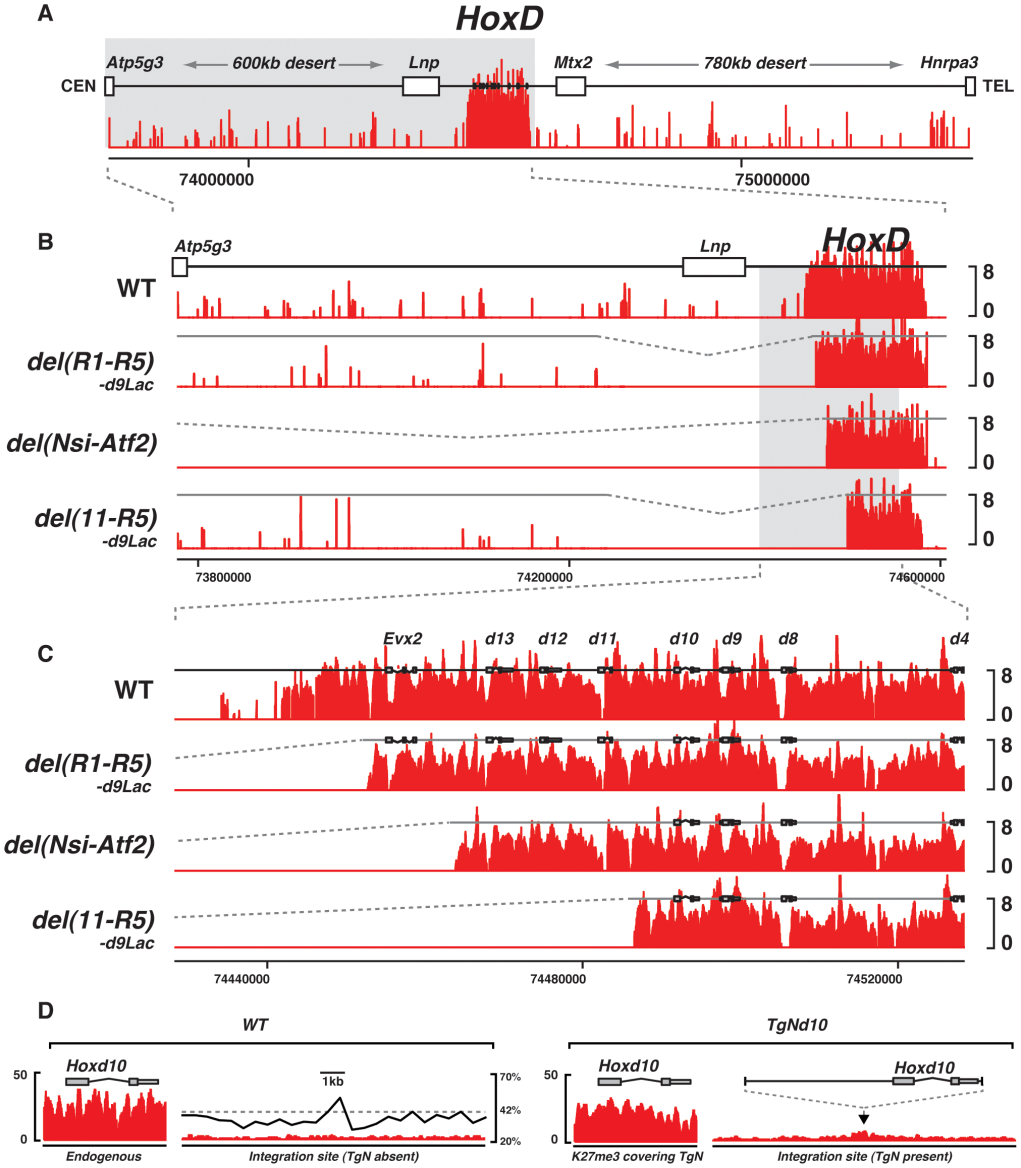
Effect of large deletions upon the H3K27me3 profiles. (A) Wild type genomic landscape of the murine *HoxD* cluster. The log2 profiles of H3K27me3 enrichment are depicted in red. (B–C) H3K27me3 profiles of either wild type, or animals harboring a deletion of the 5′ border of the H3K27me3 domain. Genotypes are specified on the left. (D) H3K27me3 profiles over the endogenous (left) and transgenic (right) *Hoxd10* sequence and over the integration site, in the presence or absence of the transgene. Wild type GC density using a 500 bp sliding window is depicted by the black line (right panel).

In the absence of any strong and discrete signal for PRC2 recognition and nucleation within the cluster itself, we asked whether Pc proteins may be targeted by elements localized within the regulatory landscapes flanking the *HoxD* cluster, which contain numerous *cis*-acting sequences. We deleted a 230 kb large piece of DNA, from eight kb upstream *Evx2* to a breakpoint located within the flanking centromeric gene desert (*del(R1-R5*)*-d9Lac*) [54] (Fig. 3B, C). While this deletion did not alter the *HoxD* gene cluster *per se*, it removed the border of the H3K27me3 domain and hence it reconstituted a neighborhood between heavily H3K27 tri-methylated nucleosomes and nucleosomes not methylated at all.

The deletion of this ‘epigenetic border’ did not elicit any loss of H3K27me3 marks over the *HoxD* cluster in the developing brain, nor did it induce any leakage over the centromeric DNA from the gene desert (Fig. 3B, C, *del(R1-R5)-d9Lac*). Therefore, as previously reported [41], the reconstitution of this artificial boundary between two chromatin domains with and without H3K27me3 marks did not lead to any spreading, at least not towards the centromeric end. We conclude that the recruitment of PRC2 is likely a sequence-specific process and that the spreading of its enzymatic activity may require some specific DNA features. The GC-content, which is unusually high within the cluster itself, while low in the sequences reconstituting the border (Fig. 2B, C), may contribute to this process.

Another mechanism to set the PcG epigenetic borders may involve transcripts encoded by the opposite DNA strand, a feature found in the *HoxA*, *HoxC* and *HoxD* clusters. In the case of *HoxD*, the *Evx2* gene is found ca. 10 kb upstream *Hoxd13* on the opposite strand. This gene, which is covered by H3K27me3 marks and locates close to the epigenetic border, was however not removed in the *del(R1-R5)-d9Lac* deletion. Therefore, we analyzed a second deletion, ca. 260 kb large, with the same upstream breakpoint into the gene desert (see above), but with a telomeric breakpoint located between *Hoxd10* and *Hoxd11*. In this *del(11-R5)-d9Lac* mutant, the entire posterior part of the *HoxD* cluster was removed including *Hoxd11*, *Hoxd12* and *Hoxd13* as well as the *Evx2* transcription unit and the epigenetic border.

In this configuration, the H3K27me3 profile remained unchanged when compared to the wild type pattern. In particular, the reconstituted epigenetic boundary was similar to that seen with the shorter *del(R1-R5)-d9Lac* deletion, suggesting that additional transcriptional units encoded by either DNA strands are not necessary for the recruitment of PRC2 at the extremity of the *HoxD* cluster, nor for the fixation of a sharp epigenetic boundary (Fig. 3B,C; *del(11-R5)-d9Lac*). In both deletions, however, a *Hoxd9*/Lac transgene was relocated at the breakpoint, raising the possibility that transgenic sequences would interfere with PRC2 recruitment and hence we used a final mutant configuration carrying a ca. 800 kb large deletion including the *HoxD* centromeric regulatory landscape. This *del(Nsi-Atf2)* deletion not only removes the 5′ epigenetic border, but also most of the regulatory elements that contact *Hoxd* genes and impose a chromatin topology to the locus [54], [55].

The H3K27me3 profile observed in such mutant brains was as in wild-type animals (Fig. 3B, C). Furthermore, ChIP-qPCR analyses revealed that the spreading of H3K27me3 from the *HoxD* cluster towards the new centromeric neighboring sequences did not exceed a 800 bp large interval, which corresponds to twice the average length of the sonicated DNA fragments (data not shown). These results further indicated that the capacity to recruit PRC2 is restricted to *Hox* genes themselves, without any contribution from the surrounding genomic sequences. To demonstrate this point, we produced a transgenic line containing the *Hoxd10* gene, which had inserted into a genomic region of average GC density and poor in H3K27me3 marks. While H3K27me3 was scored on the entire transgene, this histone modification did not spread over flanking nucleosomes, as assessed by ChIP-seq (Fig. 3D).

### *PcG* responsiveness of transgenes *in vivo*

The *Hoxd10* transgene was defined by the two *loxP* sites previously used for the deletion of this locus *in vivo* (see above). Therefore, when this transgenic stock was crossed back into a mouse carrying a homozygous deletion of *Hoxd10* (*TgN/del(10)* ^−/−^), the H3K27 trimethylation profile (or the lack thereof-) over *Hoxd10* reflected that of the ectopic *Hoxd10* copy. The *Hoxd10* locus was selected because the CpG island located upstream the promoter (CpG32 from UCSC) could be removed by using FRT sites and the Flip recombinase *in vivo*, without affecting the transcription start site (TSS). To make sure that no additional CpG islands remained after deletion of CpG32, we deleted another potential short island (CpG26) from our starting transgenic construct.

**Figure 4:**
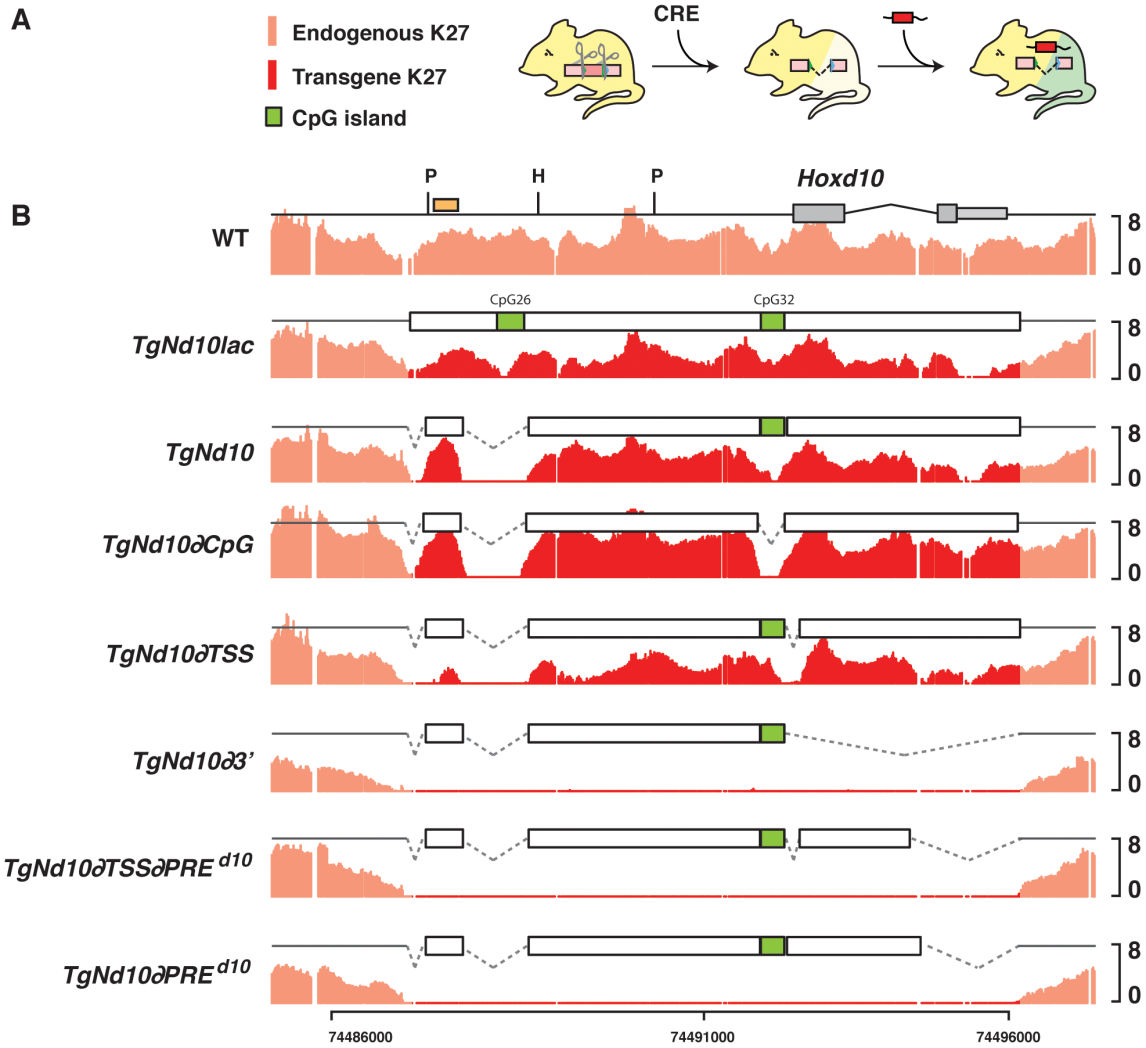
H3K27me3 profiles on transgenes *in embryo*. (A) Experimental setup, with a portions of the *Hoxd10* region injected into mice harboring a deletion of *Hoxd10* such as to distinguish between the methylation covering the endogenous locus (light red) and the transgene (dark red). (B) H3K27me3 profiles of wild type and animals carrying a transgene corresponding to the entire part -or portions thereof- of the DNA segment deleted in the *del(10)* allele. Methylation over the transgenic construct(s) is depicted in dark red. CpG islands are shown as green boxes. The orange box in the WT profile indicates the position of the probe for Southern blot in which *PvuII* (P) and *HindIII* (H) were used for digestion (see figure S3).

Transgenic animals were crossed with a Cre-deleter strain to adjust copy number to one and various transgenes were thus crossed over *Hoxd10* null mice to assess their H3K27me3 status in developing forebrains (Fig. 4A, B and Fig. S3). When the 9 kb long *Hoxd10* locus containing a *LacZ* reporter cassette was used as a transgene, H3K27 trimethylation was almost undistinguishable from wild-type littermate brains, with a strong enrichment of H3K27me3 over the entire DNA fragment (Fig. 4B, *TgNd10Lac*). Similar results were observed when the CpG26 sequences had been removed (Fig. 4B, *TgNd10*). Moreover, similar amounts of H3K27me3 were scored when the transcription start site of *TgNd10* was deleted, suggesting that the recruitment of PRC2 may be independent of transcription (Fig. 4B, *TgNd10∂TSS*) [56]. Finally, when the second CpG island was excised, the H3K27me3 profile again remained unmodified, showing that CpG rich regions are dispensable for the initial recruitment of PRC2, at least for this DNA segment and in this tissue (Fig. 4B, *TgNd10∂CpG*).

However, when a four kb large transgene containing only the 5′ sequence upstream *Hoxd10* was used, H3K27me3 marks were no longer detected, even though this construct still contained an annotated CpG island (Fig. 4B, *TgNd10∂3*) and was globally GC-rich. Of note, a transgene containing the same four kilobases together with exon 1 of *Hoxd10* showed no recruitment of PRC2 either (Fig. 4B, *TgNd10∂*PRE*^d10^*), regardless whether or not the TSS was present (Fig. 4B, *TgNd10∂TSS∂*PRE*^d10^*), suggesting that neither the TSS, nor the CpG32 are essential for recruiting PRC2 in this configuration. Mapping the insertion sites did not reveal any correlation between the presence of H3K27me3 on the transgenes and their insertion into either a H3K27me3-rich or a GC-rich DNA region. In fact, transgenes were found integrated at least 500 kb away from H3K27me3-rich spots and into DNA segments with rather average GC contents (data not shown). These experiments thus defined a 1.4 kb large DNA segment, containing exon 2 and the 3′UTR of *Hoxd10*, which was necessary for the deposition of H3K27me3 marks. This DNA segments is referred to as PRE*^d10^* below.

### PRE*^d10^* recruits PRC2 in pluripotent stem cells

**Figure 5:**
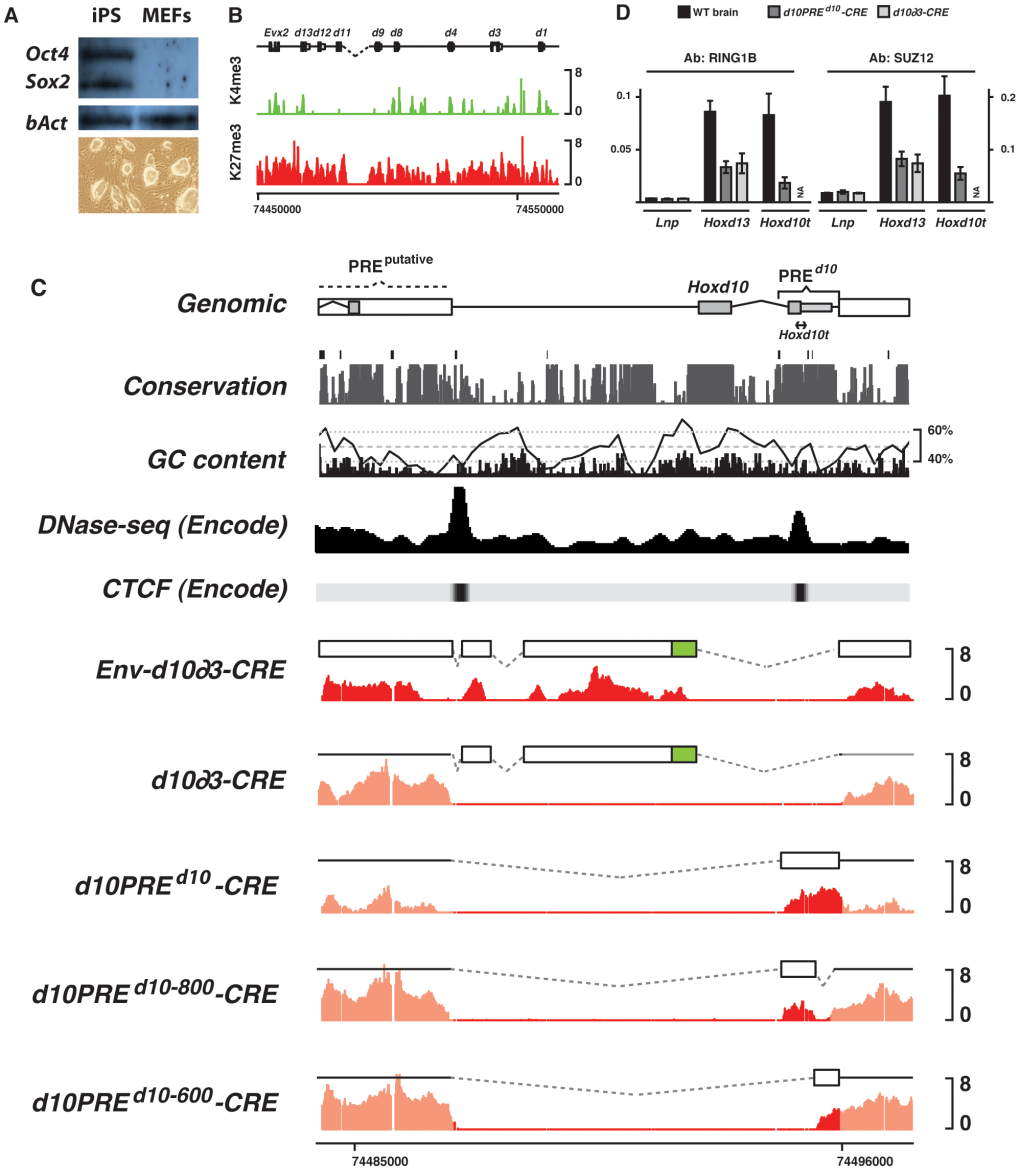
H3K27me3 profiles of transgenic constructs in iPS cells. (A) Western blot for pluripotency markers and morphology of the iPS*^del(Hoxd10)^* clone. (B) Chromatin signature of iPS cells with the observed reactivation of bivalent domains over most *Hoxd* genes. (C) H3K27me3 (red) profiles of various constructs electroporated into iPS cells carrying a deletion of *Hoxd10*. The electroporated construct is depicted by the white box above each profile. *Env-* corresponds to transgenes carrying arms for recombination, homologous to *Hoxd11* and the 3′ portion of *Hoxd10*, respectively. CRE indicates single copy integrants. Black line in the GC content panel corresponds to a running window of 200 bp. PREd10 overlaps with sites of DNase hypersensitivity and experimentally validated CTCF sites. (D) ChIP-qPCR of PRC1 (*Ring1B*) and PRC2 (*Suz12*) over various regions of the *HoxD* cluster. Consistent with the presence of H3K27me3, PRC1/2 binds randomly integrated PREd10 at levels comparable to that found in the endogenous *Hox* locus (*Hoxd13*).

While H3K27me3 marks covering the *Hox* clusters are twice as dense in differentiated tissues than in ES cells (Fig. S4 and [38], [44], [45]), the extent in coverage is identical, suggesting the implementation of the same mechanism. Consequently, we concentrated on pluripotent stem cells for further analyses of PRE*^d10^*. However, because the *HoxD* cluster is a target of polycomb repression in ES cells [38], [44], [45], we derived induced pluripotent stem (iPS) cells from mice carrying a homozygous deletion of *Hoxd10* to eliminate all endogenous signals. iPS cells are in principle indistinguishable from ES cells [57], [58] (Fig. 5A, B) and our iPS*^del(Hoxd10)^* were thus used to assess the H3K27 methylation status of distinct electroporated DNA elements, overlapping with the deleted *Hoxd10* DNA segment. Various portions of the *TgNd10* transgene were first cloned between two homologous arms (*Env*) flanking the transgenes, in the hope of comparing random and targeted integration sites. However, homologous recombination events were not found.

When either the entire *Hoxd10* fragment, including the TSS and both exons, or the 1.4 kb long PRE*^d10^* were introduced into our iPS*^del(Hoxd10)^* cells, they became H3K27 tri-methylated, in agreement with the results obtained using classical transgenesis. More surprisingly, when the 5′ sequence corresponding to that used in *TgNd10∂3* was assayed, H3K27me3 was detected too, in contrast to the results obtained in transgenic mice. We checked the capacity of either the vector backbone, or the PGK-neomycin gene promoter to recruit PRC2, by electroporating the neomycin cassette alone. The PGK promoter is ubiquitous and hence neomycin transcripts were detected in all conditions tested (Fig. S5). The gene body did not show any enrichment in H3K27me3, regardless whether cells were grown with or without G418 selection (Fig. S5).

We next assessed whether PRC2 recruitment by the *Env-d10∂3′* DNA fragment was enhanced by the presence of large concatemers of the transgene. We treated *Env-d10∂3′* cells with a CRE-expressing lentiviral construct leading to the reduction of the concatemers to a single transgene copy, devoid of selection cassette. However, after proper excision of the supernumerary transgenes, the single copy was still able to capture PRC2, even in the absence of PRE*^d10^* (Fig. 5C, *Env-d10∂3-CRE*). We verified if this recruitment was influenced by the presence of the DNA homology arms included for a potential recombination at the locus, which contained sequences from both the *Hoxd11* and a portion directly 3′ to *Hoxd10*, which could thus initiate a ‘spreading’ of H3K27me3 marks over the *Env-d10∂3* fragment. Accordingly, we electroporated *d10∂3* (42% rich in GC) into iPS*^del(Hoxd10)^* without any other surrounding DNA sequences. While H3K27me3 was detected over a multimerized version of *d10∂3*, this mark was lost after the CRE recombinase had reduced copy number to one (Fig. 5C, *d10∂3-CRE*). In contrast, when CpG-island free PRE*^d10^* (44% rich in GC) was introduced into iPS*^del(Hoxd10)^*, H3K27me3 marks were readily scored after CRE-excision of the multimers (Fig. 5C, *d10PRE^d10^-CRE*). PRC1/2 subunits were also detected over this exogenous, randomly integrated sequence, suggesting it contains all proper information necessary for PcG recruitment to ectopic sites (Fig. 5D).

To further narrow down potential PRE's within PRE*^d10^*, we split PRE*^d10^* into two smaller fragments, PRE*^d10-800^* (44% rich in GC) and PRE*^d10-600^* (43% rich in GC), which were tested as individual transgenes. Unexpectedly, both fragments were decorated by H3K27me3, when introduced into iPS*^del(Hoxd10)^* cells as single copy (Fig. 5C). This suggested that, as in *Drosophila*, mammalian polycomb recruiting elements can hardly be narrowed down to a unique sequence. Moreover, substantially less H3K27me3 was scored on either fragment, suggesting that these low interacting sequences may synergize to form a robust PRE.

## Discussion

While PREs have been relatively well identified in *Drosophila*, their existence in mammals remains restricted to some empirical examples. In this study, we show that a 1.4 kb long DNA sequence is necessary and sufficient for the recruitment of the polycomb machinery both in cultured cells and in the embryo. This sequence together with others, may be important for the silencing of this locus. However, as with the deletion of PREd11.12, the deletion of this sequence *in vivo* did not substantially affect the distribution of H3K27me3, i.e. one usual read out of *Pc* silencing, throughout the *HoxD* cluster, suggesting that this PRE may be functionally restricted to the *Hoxd10* locus.

### Necessity and robustness of *Pc* mediated silencing in *Hox* clusters

In many bilaterian species, *Hox* genes are found in one or several genomic clusters, an organization tightly associated with the necessity for these genes to properly coordinate their transcriptional activation and maintenance. In particular, animals (vertebrates or invertebrates) displaying a temporal sequence in the establishment of their segmented body plan systematically show a complete clustering of their *Hox* gene complement, whereas other animals following different strategies (such as cell lineages) usually have broken *Hox* clusters or even *Hox* genes scattered throughout the genome (refs in [59]). It was recently proposed that the temporal sequence in *Hox* gene activation was associated with the progressive removal of H3K27me3 marks [50] and that these marks helped maintaining silent genes into a repressive spatial compartment [60], [61].

This configuration may be necessary to impose a tight repression over *Hox* genes until their proper time of transcriptional activation, to avoid their precocious activity leading to homeotic transformations. In this view, the activation of the *Hox* gene family may rely upon a progressive and directional removal of the *Pc* repressive activity, which may have helped to select for gene clusters with a high density of genes and concomitant start sites and GC islands, leading to a global re-enforcement and tightening of PRC2 recruitment. A high concentration of- and co-operativity between the sequences recruiting PRC2 may readily compensate for the lack of some of them, explaining why none of our deletion mutants *in vivo* elicited a visible re-organization of the H3K27me3 profile.

### Recruiting *polycomb* complexes to DNA

Recent studies have proposed that stalled polymerase could be involved in PcG tethering [62], a proposal which could apply to the reported D11.12 PRE (48% GC) [31], since it contains an alternative start site for *HOXD11*. However, we show that transgenic constructs can lack H3K27me3 marks even though both the start sites and coding sequences are present, whereas other transgenes displayed H3K27me3 marks despite the absence of TSS. While it is possible that stalled PolII is still present at cryptic or shadowed sites, or that TSS present on some transgenes are not functional, our data do not favor the view whereby a TSS can work as a PREs. In this view, gene repression via PcG proteins likely relies on a number of regulatory mechanisms, rather than being solely due to transcriptional interference mechanism [63], [64], [65], [66], [67], [68].

As for many Drosophila PREs, PRE^d10^ overlaps with both a DNase hypersensitive site and a CTCF binding site. However, these hallmarks are present neither in the previously identified d11.12, nor in the MafB/Kreisler PREs. Moreover, it is noteworthy that, although they are both bound by PRC1 and PRC2, PRE^d10^ and PRE d11.12 are neither bound by Jarid2, nor by KDM2B, two proteins found in some PRC2 complexes to target them to appropriate loci [69], [70], [71]. It is possible that different PRE sequences throughout the *HoxD* cluster have different operational modes. Also, the presence of GC rich sequences, and more specifically their unmethylated form [70], [71], has been proposed as a pre-requisite to establish *Pc*-dependent repression due to the correlation between Polycomb group proteins and CpG islands (at least 50% GC over 200 bp) [44], [45]. Moreover, bacterial DNA sequences with high GC density are sufficient for PRC tethering in embryonic stem cells [40], [41] and two thirds of all PcG bound targets contain GC rich fragments, either in their promoters or in their gene bodies. Because of their unusually high concentration of genes, the *Hox* clusters are amongst the genomic loci with the highest GC content.

Here again however, our results do not support a high GC content as the major parameter in recruiting PRC2. We show that DNA segments with a GC content similar to the average of the mouse genome (42%) are still able to properly recruit PcG proteins and the deletion of CpG islands from our transgenic constructs did not abrogate the trimethylation of H3K27. While these results suggest that CpG islands are neither sufficient, nor required, for the tethering of PcG proteins in the context of *Hox* gene clusters, they do not rule out their potential importance for the spreading or the re-enforcement of the coverage by PRC2 (see below). The existence of CpG islands devoid of PcG, as well as PcG target DNA devoid of CpG islands, such as in the case of the first described mammalian PRE-like sequence regulating the mouse MafB/Kreisler, support this view. Moreover, sequences unable to recruit PRC when present as single copy transgenes may become H3K27me3 when concatamerized, suggesting that larger stretches of GC-high sequences can artificially recruit PRC, a possible explanation to the discrepancies observed between our results and those of others [40].

### PcG mediated repression over the *HoxD* cluster

**Figure 6:**
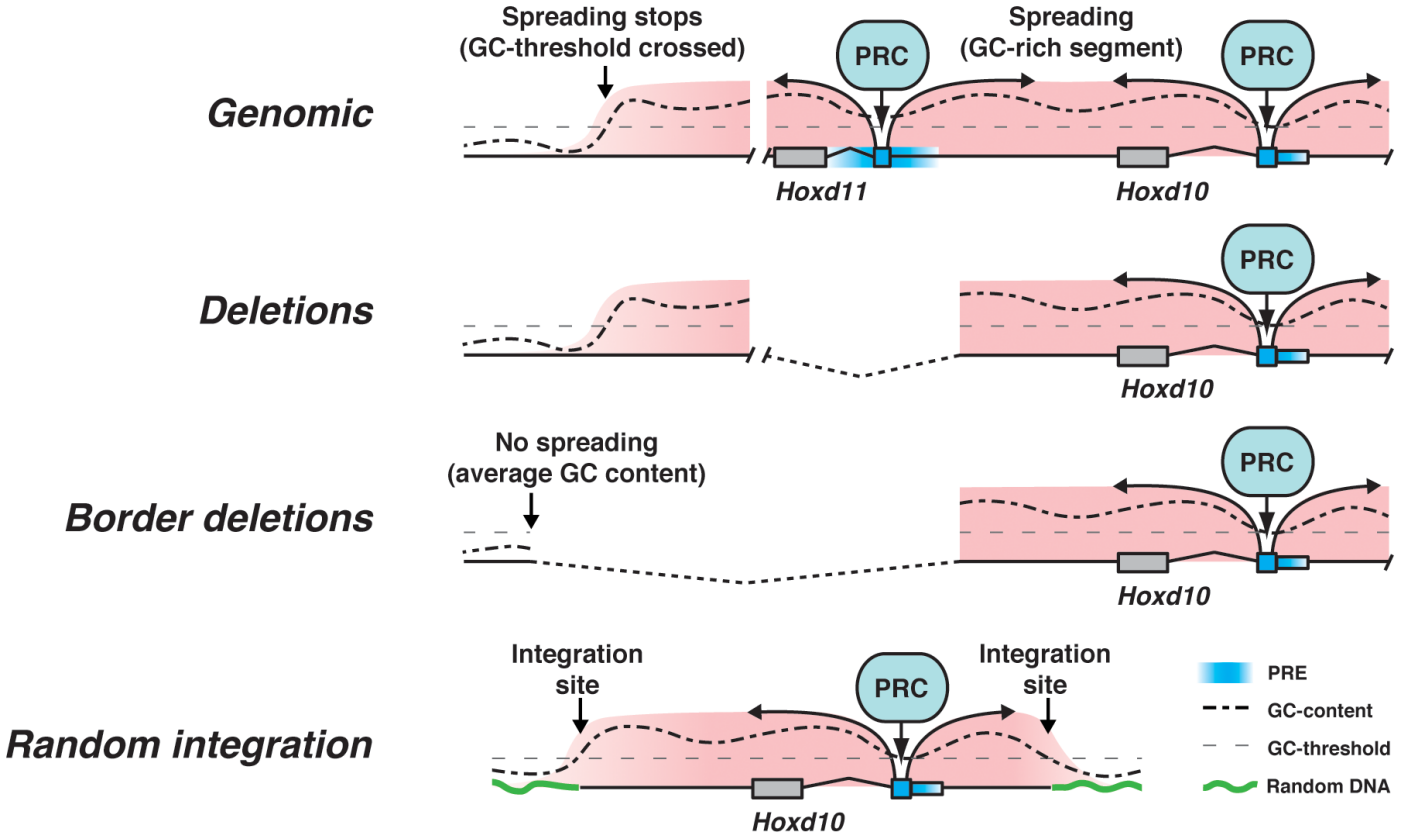
A model for PRC2 recruitment. In a first phase, PRC2 is tethered to a particular combination of low affinity PREs. Once bound, the surrounding GC density becomes important for stabilizing or strengthening the binding to target DNA. Internal deletions do not alter the general landscape. Deletion of the borders of the epigenetic domain do not lead to PcG leakage due to the translocation of the 3′ breakpoint into sequences of low or average GC density. Similar results are observed when transgenic constructs are introduced randomly into the genome.

Our data are in agreement with an *ad minima* model whereby H3K27me3 is deposited on a series of low affinity PRC2 interacting sequences, which work synergistically between themselves and together with GC-rich sequences to confer robust silencing over target genes (Fig. 6). The minimal number of such sequences required to elicit Pc-dependent silencing is unknown, as well as the mechanism underlying their cooperativity. To date, three such minimal sequences have been spotted within *HoxD*, including PRE d11.12, PRE*^d10^* and a sequence within the construct we used as homologous arms. Each *Hoxd* gene locus may thus carry at least one such sequence.

Once PRC2 tethered to low affinity PREs, the GC density may help strengthening the interaction between the repressive complexes and the surrounding DNA, either by stabilizing PRC2 or by recruiting PRC1 (Fig. 6). In agreement with this view, nucleosome density, a feature that correlates with high GC content [72], is important for the maintenance of H3K27me3, whereas PRC2 may be activated by these initial repressive marks *via* an allosteric modification [73]. Accordingly, any DNA segment, regardless of its GC content, would become H3K27 trimethylated, if introduced into the *HoxD* cluster, as is the neomycin cassette in the *rel0neo+* allele (Fig. 2C, D). Moreover, GC rich DNA segments introduced in the vicinity of a PRE could stabilize the association with the PcG complex and become H3K27 trimethylated, as in our different transgenic constructs. This view would also accommodate the absence of H3K27me3 spreading near the integration site of *TgNd10Lac*, as well as in the case of the deletion of the 5′ epigenetic border.

In such *Hox* loci, where chromatin compaction seems to be enhanced whenever the cluster is inactive, potential cross-linking artifacts may give the impression of a dense and continuous coverage by H3K27me3, whereas some regions could be devoid of PRC2. While this may indeed slightly bias the results, it remains from our genetic analyses that a range of PRE-like sequences must exist scattered within the *HoxD* cluster, instead of a few strong PRC2 airports, from which an enzymatic activity would spread, either *via* the spreading of the enzyme, or due to conformational proximity.

## Materials and Methods

### Ethics statement

All experiments involving animals were authorized and carried out according to the Swiss law on animal experimentation (LPA; No 1008/3482/0 to DD).

### Mutant mice

All stocks of mice were kept as heterozygous and bred to homozygosity. Lines were all described and can be found in previous publications of the Duboule laboratory. Two additional lines were produced by recombination between the loxP site in the second exon of *Hoxd1* and either the site telomeric to *Hoxd8* (*del(1-i)*) or the site centromeric of to *Hoxd4* (*del(1-4)*). The *del(i)* line was produced by TAMERE between the loxP telomeric of *Hoxd8* and the one centromeric to *Hoxd4*. Genotyping was performed on individual yolk sacs.

### Chromatin immunoprecipitation

Chromatin immunoprecipitation followed by quantitative polymerase chain reaction was performed as described in [74]. Briefly, cells were pre-plated 45 minutes to ensure no contamination from feeder cells. Cells or tissues were fixed for 10 and 15 minutes, respectively, in 1% formaldehyde, washed three times in cold PBS and stored at −80° before being processed using polyclonal anti-H3K27me3 antibody (Millipore, 17–622) or H3K4me3 (Millipore 17–614). ChIPped DNA was either hybridized to customized tiling arrays (see customized tiling array) or deep sequenced using the Illumina Genome Analyzer. Reads were mapped onto the mouse mm8/mm9 genome using Tophat and visualized with the integrative genome viewer and RChiV.

### Cell culture

Mouse embryonic fibroblasts were derived from heterozygous crosses of E13.5 embryos using standard protocols. Cells were cultured in standard MEF/ES cell culture conditions. MEF/ES media contained DMEM supplemented with 10% FBS and LIF (ES media only). Isolated MEF lines were first genotyped using embryonic tissues and subsequently confirmed with DNA extraction procedures. Passage three MEFs were used for iPS derivation experiments.

### Induced pluripotent stem cell derivation and manipulation

Human *Oct4*, *Klf4* and *Sox2* were cloned and separated by bacterial 2A sequences, in a single lentiviral backbone (3F). Virus was produced in 293T cells using FuGENE HD transfection reagent (Promega, E2311) and ultracentrifuged. Induced pluripotent (iPS) stem cells were derived following standard protocols [57]. Colonies were picked at d16–d18 and expanded before genotyping. Pluripotency of clones was confirmed by their ability to grow indefinitely, the expression of pluripotency markers (*SSEA1*, *Nanog*, *Oct4* and *Sox2* by immunohistochemistry and western blot, standard protocols), a non-aberrant chromosome count (by chromosome spread, standard protocols) and re-establishment of bivalent domains (K27me3 and K4me3, see ChIP).

Electroporation of induced pluripotent stem cells was performed using an Amaxa Nucleofector I and the Lonza mouse embryonic stem cell kit (Lonza VPH-1001). Briefly, 25 µg of DNA were digested overnight, phenol-chloroform purified and resuspended in 10 µl H2O. Media was changed 4 hours before electroporation. Cells were washed twice with Mg(2+)-Ca(2+)-Free PBS, trypsinized and aliquoted to 2×10^6^. Electroporated cells were plated on 10 cm dishes coated with DR4 resistant feeders. G148 selection (200 µg/ml) (Sigma G8168-10ML) was started 24 hours after electroporation and was continued until individual colonies were picked and genotyping. CRE treatment of iPS cells was done as follows: 3×10^5^ cells were plated overnight and transduced with a PGK-CRE lentiviral construct at MOI 100. Individual colonies were picked 5 days post-transduction, expanded and genotyped.

### Customized tiling array

Affymetrix custom-made tiling arrays covering two megabases surrounding the mouse *HoxD* cluster were spotted with 25-mer oligonucleotides at 15b bp resolution (Genome Assembly 2006 NCBI36/mm8: chr2:73,709,304–75,470,233). Fragmentation, labeling and hybridization of ChIPed DNA were done following standard protocols.

### Expression analysis, Southern blot and transgene mapping

Cells were first disrupted and homogenized using a Polytron (kinematic) before RNA was extracted using the RNeasy Microkit (Qiagen, 74034). qRT-PCR was performed with SYBR Green. Two biological replicates, processed in triplicates and normalized to a housekeeping gene (*Rps9*) were used to derive mean values. Primers are given in Table S1. Southern blotting was performed using standard protocols. Different probes were DIG-labeled using the PCR DIG Probe Synthesis kit (Roche, 11 636 090 910). Genomic integration mapping of transgenes/constructs was performed using the inverse PCR (iPCR) method from the Molecular Cloning Manual (third edition). Briefly, DNA was digested, phenol-chloroform precipitated, self-ligation 4 hours at room temperature and ethanol-precipitated. A first round of PCR was done with 50 ng of template before proceeding to a second round of PCR, using nested primers. Finally, distinct amplicons were purified using the QIAquick Gel Extraction kit (Qiagen, 28704) and sent for sequencing.

### Data analysis

Raw hybridization data was extracted using the two-sample comparison analysis and quantile normalized using Tiling Analysis Software (TAS) from Affymetrix. Data was exported as plain text using a log2 or −10log10 scale for the signal, respectively the p-value. Files were visualized in RChiV, an in-house developed genome browser, which takes into account the deleted segment and normalizes the signal using a sliding window approach.

## Supporting Information

Figure S1 Binding profiles of PRC1 and PRC2 over the *HoxD* cluster in mutant configurations. (A) ChIP-qPCR profiles of PRC1 (*Ring1B*, upper panel) and PRC2 (*Ezh2* (middle panel) and *Suz12* (lower panel)) over the *HoxD* cluster. The wild type values of six genes (from *Evx2* to *Hoxd3*) found within the H3K27me3 domain are used as positive controls (black). *Lnp* and *Mtx*2 are found outside of the H3K27me3 domain and are thus used as negative controls (black). Different mutant configurations are color coded and specified on the top (*del(10*), *del(10-11)*, *del(9-12)*, *del(10-13)*, *del(11-R5)*). *dN* stands for *HoxdN*. NA refers to the absence of the given DNA segment in the specified allele. NI refers to mutant alleles which where not included in the experiment (*Suz12* on both *del(10-11)* and *del(11-R5)*).
(TIF)

Figure S2 Effect of large deletions upon the H3K27me3 profiles. (A) Wild type genomic landscape of the murine *HoxD* cluster and flanking gene deserts. (B) H3K27me3 profiles of wild type and deleted animals. Genotypes are specified on the left.
(TIF)

Figure S3 Southern blot of transgenic animals. Southern blot using a *Hoxd10* specific probe (see Figure 4B). Restriction enzymes used for the experiments are specified below and mutant strains are on the top. Wild type bands are depicted by the black arrows whereas transgenic fragments are shown in red. The founders (+/−) exhibit two bands before being crossed over a *Hoxd10* deletion, where only the transgenic band remains (−/−). A wild type sample (+/+) was used as control (right panel). The positions of both the restriction sites for *PvuII* and *HindIII* and the probe used for southern blot are depicted on the wild type profile in Fig. 4B.
(TIF)

Figure S4 H3K27me3 profiles in pluripotent and terminally differentiated cells. A large (A) or focused (B) view of wild type H3K27me3 profiles from differentiated cells dissected from the embryonic brain (top) compared to pluripotent cells derived from a *del(10)* embryo (iPS, bottom).
(TIF)

Figure S5 H3K27me3 and RNA profiles in various cell lines. ChIP-qPCR and mRNA expression of control and *Hoxd* genes in various constructs eletroporated in iPS cells carrying a deletion of *Hoxd10*. *Lnp* is located outside the *HoxD* cluster and is used as a control for active genes, while *Hoxd13* is used as a control for silent genes. Clones and culture conditions are color coded and specified on the top. G418 stands for the presence (+) or absence (−) of the antibiotic. Vector refers to a control cell line.
(TIF)

Table S1 List of the primers used for RT-PCR, either for ChIP experiments (top) or for RNA dosage (bottom).
(DOC)
