# Supplementary figures and images for: A Genetic Approach to the Recruitment of PRC2 at the HoxD Locus
Source: PLoS Genet. 2013 Nov 7;9(11):e1003951. doi: 10.1371/journal.pgen.1003951 (PMC3820793; doi:10.1371/journal.pgen.1003951)

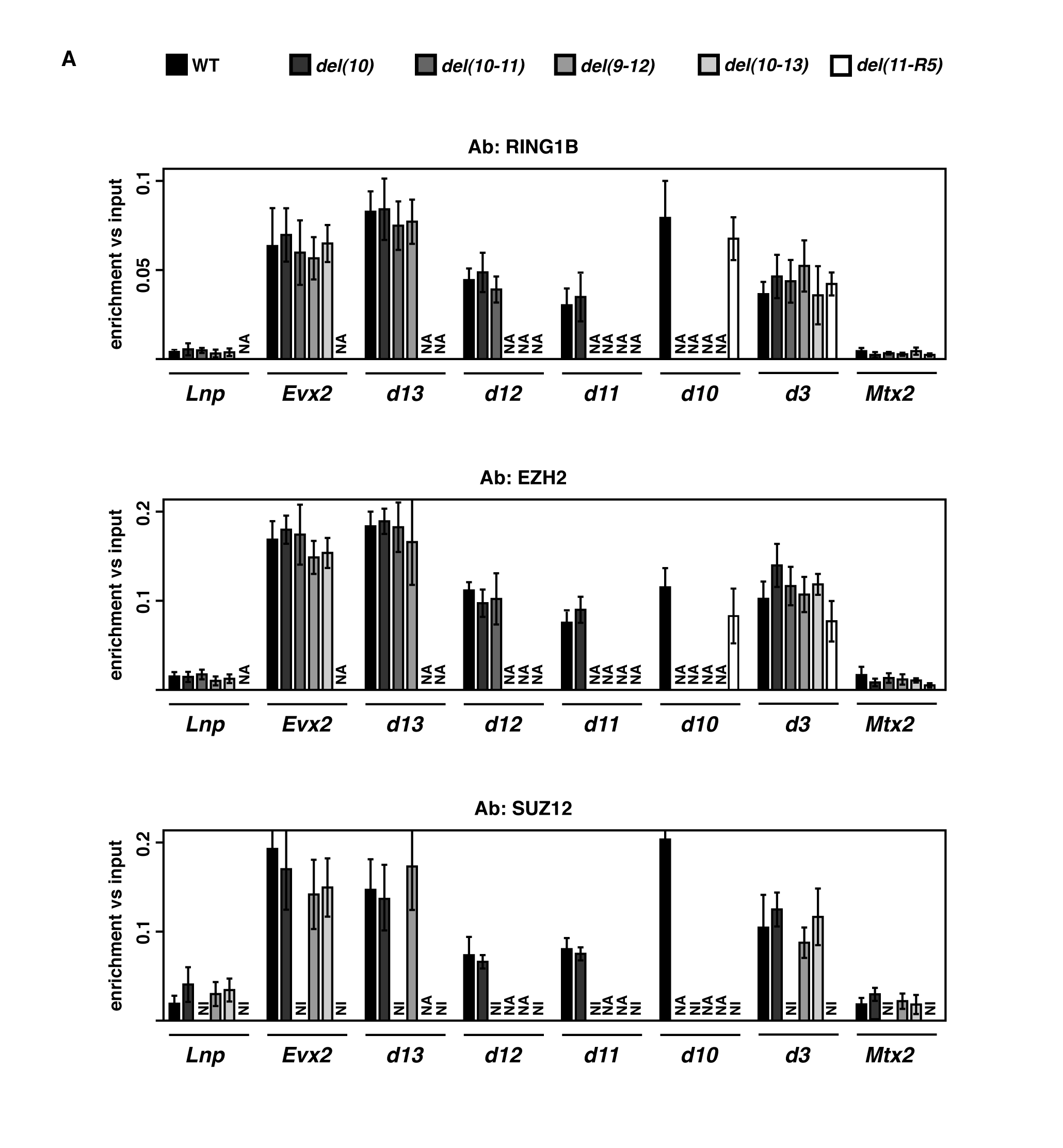

Supplement: Figure S1 — Binding profiles of PRC1 and PRC2 over the HoxD cluster in mutant configurations. (A) ChIP-qPCR profiles of PRC1 (Ring1B, upper panel) and PRC2 (Ezh2 (middle panel) and Suz12 (lower panel)) over the HoxD cluster. The wild type values of six genes (from Evx2 to Hoxd3) found within the H3K27me3 domain are used as positive controls (black). Lnp and Mtx2 are found outside of the H3K27me3 domain and are thus used as negative controls (black). Different mutant configurations are color coded and specified on the top (del(10), del(10-11), del(9-12), del(10-13), del(11-R5)). dN stands for HoxdN. NA refers to the absence of the given DNA segment in the specified allele. NI refers to mutant alleles which where not included in the experiment (Suz12 on both del(10-11) and del(11-R5)). (TIF) [file pgen.1003951.s001.tif]

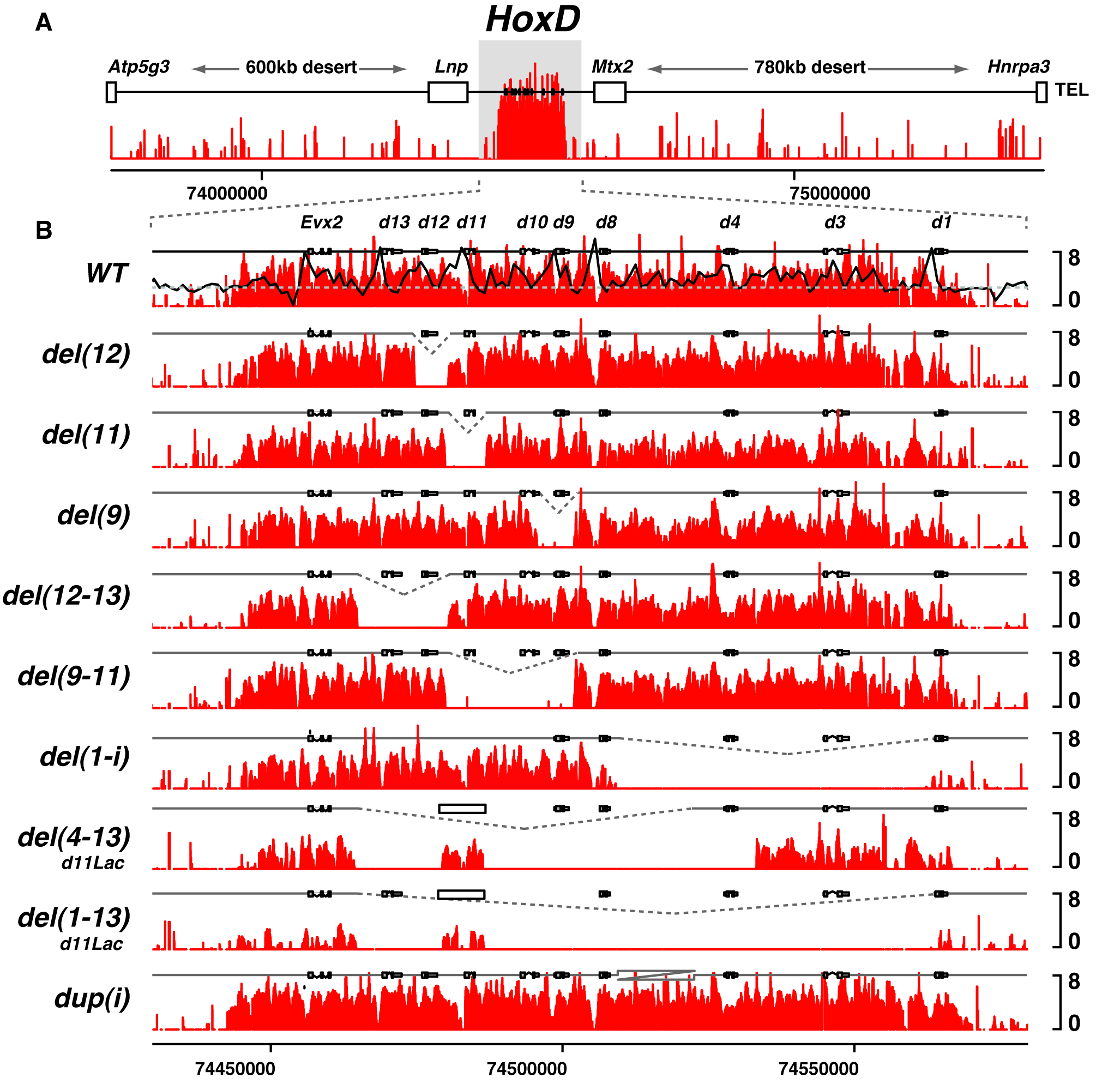

Supplement: Figure S2 — Effect of large deletions upon the H3K27me3 profiles. (A) Wild type genomic landscape of the murine HoxD cluster and flanking gene deserts. (B) H3K27me3 profiles of wild type and deleted animals. Genotypes are specified on the left. (TIF) [file pgen.1003951.s002.tif]

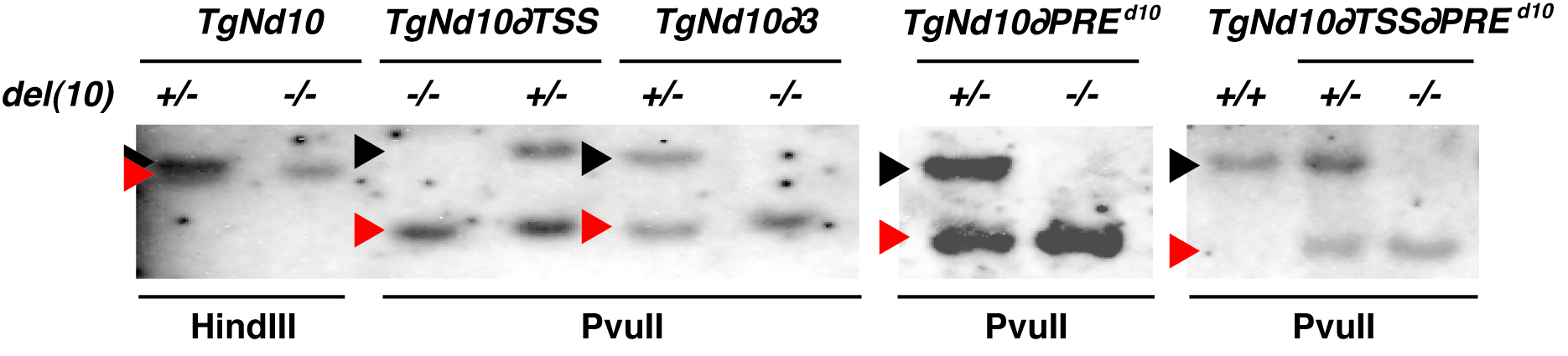

Supplement: Figure S3 — Southern blot of transgenic animals. Southern blot using a Hoxd10 specific probe (see Figure 4B). Restriction enzymes used for the experiments are specified below and mutant strains are on the top. Wild type bands are depicted by the black arrows whereas transgenic fragments are shown in red. The founders (+/−) exhibit two bands before being crossed over a Hoxd10 deletion, where only the transgenic band remains (−/−). A wild type sample (+/+) was used as control (right panel). The positions of both the restriction sites for PvuII and HindIII and the probe used for southern blot are depicted on the wild type profile in Fig. 4B. (TIF) [file pgen.1003951.s003.tif]

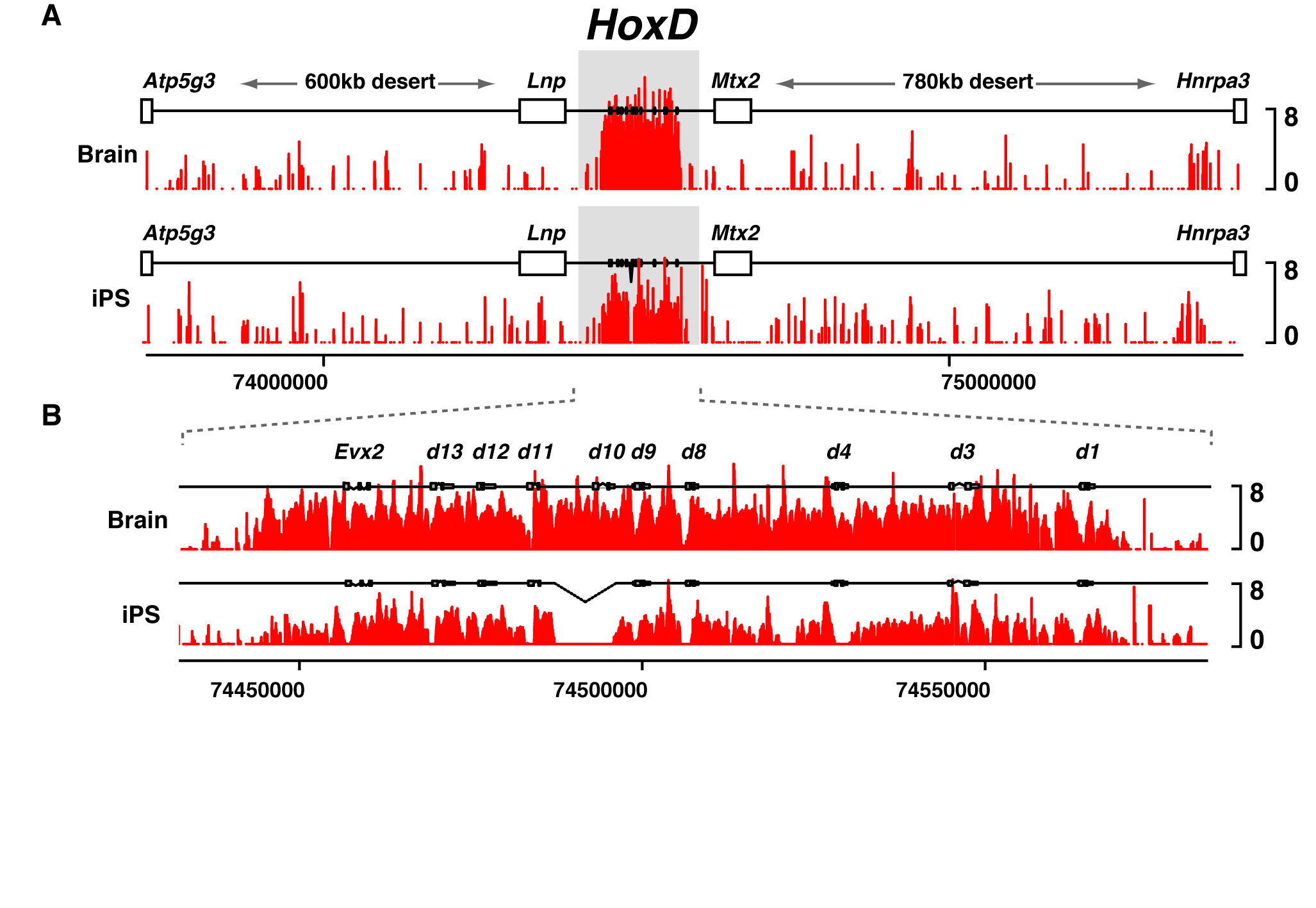

Supplement: Figure S4 — H3K27me3 profiles in pluripotent and terminally differentiated cells. A large (A) or focused (B) view of wild type H3K27me3 profiles from differentiated cells dissected from the embryonic brain (top) compared to pluripotent cells derived from a del(10) embryo (iPS, bottom). (TIF) [file pgen.1003951.s004.tif]

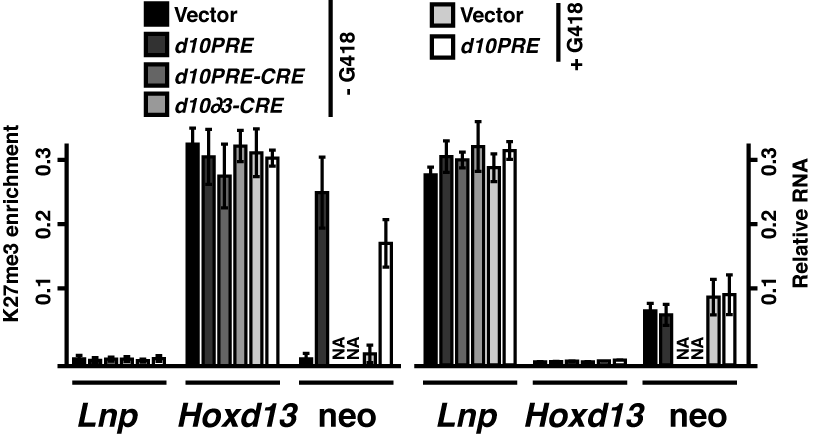

Supplement: Figure S5 — H3K27me3 and RNA profiles in various cell lines. ChIP-qPCR and mRNA expression of control and Hoxd genes in various constructs eletroporated in iPS cells carrying a deletion of Hoxd10. Lnp is located outside the HoxD cluster and is used as a control for active genes, while Hoxd13 is used as a control for silent genes. Clones and culture conditions are color coded and specified on the top. G418 stands for the presence (+) or absence (−) of the antibiotic. Vector refers to a control cell line. (TIF) [file pgen.1003951.s005.tif]
