# Supplementary material for: A Genetic Approach to the Recruitment of PRC2 at the HoxD Locus
Source: PLoS Genet. 2013 Nov 7;9(11):e1003951. doi: 10.1371/journal.pgen.1003951 (PMC3820793; doi:10.1371/journal.pgen.1003951)
Supplement: Table S1 — List of the primers used for RT-PCR, either for ChIP experiments (top) or for RNA dosage (bottom). (DOC) [file pgen.1003951.s006.doc]

**Supplementary Table 1.**

**List of RT-PCR primers used for ChIP and RNA detection.**

|  | Forward | Reverse |
| --- | --- | --- |
| 74807700 | aaagcgtgaccccggttc | tgcgctttgacaatcctagtg |
| 74785800 | tatcaaaccccgcatgacaa | ctgagaagccgcagtttgg |
| Mtx2 | cctcttgaagctgtttctgtcttg | ttgattgcaacctttcagcag |
| Hoxd3 | ccaggcgcgtcctcc | ggatccttgcggctgatttat |
| Hoxd8ex1 | ggcgaggccatcaatcc | gacctcgggtgcaaaatgac |
| Hoxd10-prom | ttttcttctggcccggtttt | aactctgatccccggactcc |
| Hoxd10t | ccttgtagcctttccttgtg | tacacatgcgaccagaac |
| Hoxd11ex1 | ttctacagcgccgtgggt | gaactgatcaaagccctggg |
| Hoxd12ex1 | cctccagcttcaaggaagaca | ccccggccacttgca |
| Hoxd13ex1 | cttacagcagaacgctctcaagtc | gtacttctccaccgggaaacc |
| Lnp | cagcctacactagcgcggag | ggtgtggcaggaggatttag |
| β-actin promoter | gcaggcctagtaaccgagaca | agttttggcgatgggtgct |
| β-actin coding | tcctggcctcactgtccac | gtccgcctagaagcacttgc |
| Oct4 | acatcgccaatcagcttgg | agaaccatactcgaaccacatcc |
| Oct4 promoter | ggctctccagaggatggctgag | tcggatgccccatcgca |
| Oct4 coding | cctgcagaaggagctagaaca | tgtggagaagcagctcctaag |
| Sox2 | acagatgcaaccgatgcacc | tggagttgtactgcagggcg |
| Sox2 promoter | ccatccacccttatgtatccaag | cgaaggaagtgggtaaacagcac |
| Sox2 coding | ggagcaacggcagcta | gtagcggtgcatcggt |

|  |  |  |
| --- | --- | --- |
| lnp-ex2/3 | ccttggatcccctgctacttc | ggagaatgggtcttgctaaaggt |
| hoxd13–ex1/2 | ggtgtactgtgccaaggatcag | cacatgtccggctggttt |
| neomycin | gcgcccggttctttttg | cctcgtcctgcagttcattca |
